# Understanding the pharmacokinetic journey of Fc-fusion protein, rhIL-7-hyFc using complementary approach of two analytical methods, accelerator mass spectrometry and ELISA

**DOI:** 10.1093/abt/tbae004

**Published:** 2024-02-06

**Authors:** Anhye Kim, Min-Seok Oh, Gwan-Ho Lee, Seongeun Song, Mi-sun Byun, Donghoon Choi, Byung-Yong Yu, Howard Lee

**Affiliations:** Department of Clinical Pharmacology and Therapeutics, CHA Bundang Medical Center, CHA University, Seongnam 13496, Republic of Korea; Department of Biomedical Informatics, CHA University School of Medicine, CHA University, Seongnam 13488, Republic of Korea; Institute for Biomedical Informatics, CHA University School of Medicine, CHA University, Seongnam 13488, Republic of Korea; Research Resources Division, Advanced Analysis and Data Center, Korea Institute of Science and Technology, Seoul 02792, Republic of Korea; Department of Stem Cell Biology, School of Medicine, Konkuk University, Seoul 05029, Republic of Korea; Research Resources Division, Advanced Analysis and Data Center, Korea Institute of Science and Technology, Seoul 02792, Republic of Korea; Research Resources Division, Advanced Analysis and Data Center, Korea Institute of Science and Technology, Seoul 02792, Republic of Korea; Department of Pharmacy, Yonsei Institute of Pharmaceutical Sciences, Yonsei University, Incheon 21983, Republic of Korea; Clinical Development Division, Genexine, Inc., Seoul 07789, Republic of Korea; Research Institute, NeoImmuneTech, co. Ltd., Pohang 37666, Republic of Korea; Research Resources Division, Advanced Analysis and Data Center, Korea Institute of Science and Technology, Seoul 02792, Republic of Korea; Department of Molecular Medicine and Biopharmaceutical Sciences, Graduate School of Convergence Science and Technology, Seoul National University, Seoul 08826, Republic of Korea; Department of Clinical Pharmacology and Therapeutics, Seoul National University Hospital, Seoul 03080, Republic of Korea; Advanced Institute of Convergence Technology, Suwon 16229, Republic of Korea

**Keywords:** antibody-based therapeutics, Fc-fusion protein, IL-7, ELISA, accelerator mass spectrometry, pharmacokinetics

## Abstract

Antibody-based therapeutics (ABTs), including monoclonal/polyclonal antibodies and fragment crystallizable region (Fc)-fusion proteins, are increasingly used in disease treatment, driving the global market growth. Understanding the pharmacokinetic (PK) properties of ABTs is crucial for their clinical effectiveness. This study investigated the PK profile and tissue distribution of efineptakin alfa, a long-acting recombinant human interleukin-7 (rhIL-7-hyFc), using enzyme-linked immunosorbent assay (ELISA) and accelerator mass spectrometry (AMS). Totally, four rats were injected intramuscularly with 1 mg/kg of rhIL-7-hyFc containing ^14^C-rhIL-7-hyFc, which was prepared via reductive methylation. Serum total radioactivity (TRA) and serum rhIL-7-hyFc concentrations were quantified using AMS and ELISA, respectively. The TRA concentrations in organs were determined by AMS. Serum TRA peaked at 10 hours with a terminal half-life of 40 hours. The rhIL-7-hyFc exhibited a mean peak concentration at around 17 hours and a rapid elimination with a half-life of 12.3 hours. Peak concentration and area under the curve of TRA were higher than those of rhIL-7-hyFc. Tissue distribution analysis showed an elevated TRA concentrations in lymph nodes, kidneys, and spleen, indicating rhIL-7-hyFc’s affinity for these organs. The study also simulated the positions of 14C labeling in rhIL-7-hyFc, identifying specific residues in the fragment of rhIL-7 portion, and provided the explanation of distinct analytes targeted by each method. Combining ELISA and AMS provided advantages by offering sensitivity and specificity for quantification as well as enabling the identification of analyte forms. The integrated use of ELISA and AMS offers valuable insights for the development and optimization of ABT.

## Teaser

Statement of Significance: The pharmacokinetic (PK) journey of Efineptakin alfa, a long-acting recombinant human interleukin-7, was explored through the complementary use of two bioanalytical methods, ELISA and AMS. This integrated approach enhanced comprehension of its PK fate, encompassing PK profiles and tissue distribution, and demonstrated its promising potential for the antibody-based therapeutics development.

## INTRODUCTION

Antibody-based therapeutics (ABTs) have evolved as new treatments of various diseases in oncology, immunology, hematology, endocrinology, and infection since the first monoclonal antibody (mAb) was introduced to the global pharmaceutical market several decades ago. The value of global market for ABT is forecasted to grow explosively and to generate a revenue of US$300 billion by 2025 [1, 2]. This has been enabled by the advent of high-technologies, which led to development of the various types of engineered ABT such as therapeutic mAb, bispecific antibody, antibody–drug conjugate (ADC), and Fc-fusion protein. To enhance the clinical success rate of ABTs development in the future, it is necessary to optimize the efficacy based on the elaborate pharmacokinetic (PK)/pharmacodynamic (PD) properties [3, 4].

In order to interpret and optimize the PK properties [5] of ABTs, special considerations should be given not only to their diverse structures and unique ADME profiles but also to the bioanalytical methods and the various forms of analytes. In the case of mAbs consisting of the major portion in ABTs market, each component of the structure has different targets, i.e., fragment antigen-binding region (Fab) for antigen recognition and fragment crystallizable region (Fc) for immune cell engagement or improved biodistribution. For various types of ABTs, including mAbs and Fc-fusion proteins, ligand-binding assays are the predominant and robust analytical methods for PK/PD and safety assessments. These biotherapeutics can exist in various forms *in vivo*: free, partially free, bound and total forms, and so on [6]. Depending on the bioanalytical methods, the type of quantifiable analytes may vary. For example, if a human Fc-specific mAb adopts an enzyme-linked immunosorbent assay (ELISA) to bind human Fc, it can recognize only analyte forms containing Fc even though analytes having only Fab portions that also exist in serum, and this information is more relevant to understand the PK/PD of the mAb. Thus, additional bioanalytical methods such as mass spectrometry may improve the selectivity, precision, and accuracy, which are better suited for detecting analytes having various forms existing in the body or anti-drug antibody (ADA) [3].

Efineptakin alfa, an Fc-fused long-acting recombinant human interleukin-7 (rhIL-7-hyFc), is currently under clinical development to proliferate and differentiate T cells in patients with immune deficiency of various causes [7]. rhIL-7-hyFc is a homodimeric human IL-7 fused to the immunoglobulin D/G4, which helps the entity stay longer in the body [7, 8]. In the phase 1 study with rhIL-7-hyFc, serum concentrations of IL-7 were determined using a commercial human IL-7 ELISA kit [7]. This bioanalytical assay, however, was not best suited to adjust for the baseline IL-7 level. To investigate the utility of additional bioanalytical methods, we conducted a non-clinical study in rats after a single intramuscular (IM) administration of ^14^C-rhIL-7-hyFc and evaluated the PK of total radioactivity (TRA) using accelerator mass spectrometry (AMS) and serum IL-7 concentration measure by ELISA. By adding the analysis of TRA in various tissues, we also aimed to explore the disposition characteristics of rhIL-7-hyFc.

## MATERIALS AND METHODS

### Production of ^14^C-rhIL-7-hyFc

^14^C-rhIL-7-hyFc was prepared via reductive methylation of free lysine residues of rhIL-7-hyFc using ^14^C-formaldehyde. A diluted solution of rhIL-7-hyFc (0.5 mg/ml) was prepared by adding 0.4 ml of rhIL-7-hyFc (25 mg/ml) to 19.6 ml of preparation buffer. To prepare ^14^C-rhIL-7-hyFc, 1.733 ml of ^14^C-formaldehyde solution (0.001%) was added to the diluted rhIL-7-hyFc solution, and 0.2 ml of 1.0 M NaBH_3_CN (Merck, Darmstadt, Germany) was added; 0.2 ml of 1.0 M NaBH_3_CN was repeatedly added three times at 10-minute intervals and then the reaction was terminated after adding 0.433 ml of 10% glycine. The reaction solution was concentrated at 3500 × g at 4°C for 20 minutes using a 30 kDa centrifugal concentrator. The ^14^C- rhIL-7-hyFc solution was desalted using a pre-equilibrated Sephadex G-25, disposable PD 10 Desalting column (Cytiva, Grens, Switzerland).

Assessment of ^14^C-rhIL-7-hyFc, including protein quantification, chemical purity, biological activity, and other parameters, was proceeded according to the Regulation on Review and Authorization of Biological Products published by the Ministry of Food and Drug Safety of the Republic of Korea. ^14^C-rhIL-7-hyFc was verified that it adhered to the evaluation criteria established for rhIL-7-hyFc, an original product.

### Non-clinical study for PK analysis and tissue distribution of ^14^C-rhIL-7-hyFc

Four male Sprague–Dawley rats (ORIENT Bio Laboratory Animal, Sung-Nam, Korea) were studied, and their age at dosing and average weight were 7 weeks and 0.22 kg, respectively. All experimental procedures were performed according to the guidelines and with the approval of the Institutional Animal Care and Use Committee (IACUC) of the Korea Institute of Science and Technology (KIST) (IACUC number: KIST-2019-022). The dose and route of administration of ^14^C-rhIL-7-hyFc were decided as a single IM injection of 1 mg/kg (162 μg/kg as human equivalence dose) by taking into account the concentration range, which can be determined with sufficient sensitivity of the analytic methods adopted in this study. The dosing formulation was prepared for a target rat dose of 1 mg/kg of rhIL-7-hyFc by mixing of 0.0055 mg of ^14^C-rhIL-7-hyFc containing a radioactivity of 0.3285 kBq (8.8878 nCi) and unlabeled rhIL-7-hyFc at ~ 0.216 mg. The IP solutions (specific activity (SA), 1.49 kBq/mg or 40.16 nCi/mg) of 0.30 ~ 0.36 kBq (8.06 ~ 9.74 nCi) per rat were injected intramuscularly in the thigh muscles of the right hind limb. The blood samples for PK analysis were collected from the catheter inserted in the right jugular vein at 0 (pre-dose), 1, 4, 10, 24, 48, 72, 96, 120, and 168 hours post-dose. After completion of blood sampling, the target organs, including immune-related organs (thymus, lymph node, spleen, and bone marrow) and major elimination organs (liver and kidney), were separated, weighed, and homogenized for each rat.

### Determination of serum concentration of rhIL-7-hyFc using ELISA

Serum IL-7 concentrations were determined using the Quantikine HS ELISA Human IL-7 immunoassay kit (R&D Systems, Minneapolis, MN). The lower limit of quantitation was 0.78 ng/ml, and the calibration curve was linear over the concentration range of 0.78–50.0 ng/ml. The accuracy ranged 83.8–116.9% and the precision was ≤ 11.2% (coefficient of variation).

### Graphitization of samples and AMS measurement

To analyze the TRA in serum and organ samples, 25 and 20–50 μl (1 mg carbon equivalent) of serum and organ samples (spleen, liver, kidney, lymph nodes, thymus, and bone marrow) were prepared, respectively, and were dried using a vacuum concentrator (LABCONCO, Kansas City, Mo, USA). To proceed with oxidation, copper oxide (600 mg) was added to the sample tube and then the sample tube was transferred to the oxidation quartz tube. The sealed quartz tube was reacted for 3 hours in a 900°C furnace (JISICO, Seoul, South Korea). The oxidized gas (carbon dioxide) was transported to the on-site reduction tube through liquid nitrogen (LN_2_) trapping by a transport system manufactured by the KIST; the non-condensable gas at liquid nitrogen temperature (−196°C) was removed under vacuum, and the carbon dioxide is reacted at 530°C for 6 hours to form graphite under the iron catalyst. The graphite generated on iron surface was mixed using a metal rod and was then pressed with a KIST pressing tool to create an AMS measurement target. KIST AMS is a 6MV Tandentron (HVEE, High Voltage Engineering Europa, BV, the Netherlands) with a terminal voltage set to 3 MV to accelerate the carbon atomic ions (C^−^); the system background was ^14^C/^12^C = ~ 5$\times$10^−16^ (as determined using pyrolytic graphite sheet). SRM 4990C (1.3407 modern carbon (MC); Oxalic acid II) standard sample provided by National Institute of Standards and Technology (NIST) was prepared and was measured for calibration of AMS samples. C3 (1.2941 MC, cellulose) and C8 (0.1503 MC, oxalic acid) were prepared as reference materials (obtained from the International Atomic Energy Agency) for the confirming sample treatment and AMS measurement process. In general, at least 50 000 “counts” were scored to obtain under −1% inaccuracy. The ^14^C/^12^C ratios in each sample were normalized to the Oxalic acid II results and the final MC value was calculated.

### Quantification of TRA (^14^C-rhIL-7-hyFc) by AMS analysis

To investigate *in vivo* TRA, ^14^C/^12^C ratio was calculated in the serum and targeted organ using AMS. TRA concentration, amount, and % ID/g containing intact rhIL-7-hyFc and rhIL-7-hyFc fragments were calculated using ^14^C/^12^C ratio and SA. The measurement results of AMS were obtained by the ratio of the amount of ^14^C (^14^C_rhIL-7-hyFc_ and ^14^C_sample_) and ^12^C (^12^C_sample_) as shown in the following equation Eq. (1). *G*_mesu_ and *G*_blank_ are the value of ^14^C/^12^C of the sample and blank, respectively. ^12^C_sample_ is the amounts of carbon (^12^C) contained in the prepared sample. *G*_sample_ was calculated by subtracting *G*_blank_ from *G*_mesu_, as shown in Eq. (2).


(1) \begin{equation*} {G}_{mesu}=\frac{{{}^{14}C}_{hIL-7- hyFc}+{{}^{14}C}_{sample}}{{{}^{12}C}_{sample}},{G}_{blank}=\frac{{{}^{14}C}_{sample}}{{{}^{12}C}_{sample}}, \end{equation*}

(2) \begin{equation*} {G}_{sample}(MC)={G}_{mesu}(MC)-{G}_{blank}(MC) \end{equation*}

To proceed with the quantification of TRA using AMS, the MC value was converted into Curie (Ci) units as shown in Eq (3). The ${G}_{hIL-7- hyFc}$ present in ${G}_{\mathrm{sample}}$ was then calculated by applying the SA value to the converted measured value using Eq. (4).


(3) \begin{equation*} {G}_{\mathrm{sample}}\ \left(f\mathrm{Ci}\right)={G}_{\mathrm{sample}}\left(\mathrm{MC}\right)\times 6.108\ f\mathrm{Ci}/\mathrm{mgC}, \end{equation*}

(4) \begin{align*} &{G}_{hIL-7- hyFc}={G}_{\mathrm{sample}}\ \left(f\mathrm{Ci}\right)\div 4.016\times{10}^7\ f\mathrm{Ci}/\mathrm{mg}\nonumber\\& \left(\mathrm{Specific}\ \mathrm{Activity}\right). \end{align*}

Serum TRA concentration (ng/ml) was ${G}_{hIL-7- hyFc}$ divided by the volume of the sample (${V}_{\mathrm{sample}}$) as shown in Eq. (5).


(5) \begin{align*} &\mathrm{Serum}\ \mathrm{TRA}\ \mathrm{concentration}\ \left(\mathrm{ng}/\mathrm{ml}\right)\nonumber\\&\qquad ={G}_{\mathrm{hIL}-7-\mathrm{hyFc}}\times \frac{1}{V_{\mathrm{sample}}\left(\mathrm{ml}\right)}. \end{align*}

Organ TRA concentration (pg/mg) and amount ($\mathrm{\mu} \mathrm{g}$) were ${G}_{\mathrm{hIL}-7-\mathrm{hyFc}}$ divided by the weight of the sample (${W}_{\mathrm{sample}}$) and the ratio of the total weight of the organ (*W*_organ_) to *W*_sample_ as shown in Eqs. (6) and (7), respectively.


(6) \begin{align*} &\mathrm{Organ}\ \mathrm{TRA}\ \mathrm{concentration}\ \left(\mathrm{pg}/\mathrm{mg}\right)\nonumber\\ &={G}_{\mathrm{hIL}-7-\mathrm{hyFc}}\times \frac{1}{W_{\mathrm{sample}}\left(\mathrm{mg}\right)}, \end{align*}

(7) \begin{align*} \mathrm{Organ}\ \mathrm{TRA}\ \mathrm{amount}\ \left(\mathrm{\mu} \mathrm{g}\right)=&\ {G}_{\mathrm{hIL}-7-\mathrm{hyFc}}\nonumber\\ &\times \frac{W_{\mathrm{organ}}\left(\mathrm{mg}/\mathrm{organ}\right)}{W_{sample}(mg)}\ . \end{align*}

Organ TRA uptake was calculated from the organ TRA concentration converted in the μg/g unit (Eq. (8)) and was then divided by rhIL-7-hyFc injection dose $\left({D}_{\mathrm{hIL}-7-\mathrm{hyFc}}\right)$as shown in Eq (9).


(8) \begin{align*} &\mathrm{Organ}\ \mathrm{TRA}\ \mathrm{concentration}\ \left(\mathrm{\mu} \mathrm{g}/\mathrm{g}\right)\nonumber\\ &=\ \mathrm{Organ}\ \mathrm{TRA}\ \mathrm{concentration}\ \left(\mathrm{pg}/\mathrm{mg}\right)\times \frac{\mathrm{\mu} \mathrm{g}}{\mathrm{pg}}\nonumber\\ &\quad\ \times \frac{\mathrm{mg}}{\mathrm{g}}\times \frac{1}{1000}, \end{align*}

(9) \begin{align*} &\mathrm{Organ}\ \mathrm{TRA}\ \mathrm{uptake}\ \left(\%\mathrm{ID}/\mathrm{g}\right)\nonumber\\ &=\mathrm{Organ}\ \mathrm{TRA}\ \mathrm{concentration}\ \left(\mathrm{\mu} \mathrm{g}/\mathrm{g}\right)\qquad\quad\ \ \nonumber\\ &\quad\div{D}_{\mathrm{hIL}-7-\mathrm{hyFc}}\ \left(\mathrm{\mu} \mathrm{g}\right)\times 100\ . \end{align*}

### PK analysis

To derive the PK parameters of TRA and serum rhIL-7-hyFc, a non-compartmental method was used in the Phoenix WinNolin (version 8.3.5, Certara, St Louis, MO, USA). The observed values were used for the initial peak concentration (*C*_max_) and time to reach at *C*_max_ (*T*_max_) of the analytes. The area under the serum concentration–time curve (AUC) from zero to the last measurable concentration (AUC_last_) was calculated using the linear trapezoidal rule. In addition, AUC from time to infinity (AUC_inf_) was estimated by the sum of AUC_last_ and the last measurable concentration divided by the slope of the regression line between the logarithmically transformed concentrations and time.

### Simulation of ^14^C labeling position

^14^C-rhIL-7-hyFc was synthesized by labeling the ^14^C-methyl group at the L-lysine position of rhIL-7-hyFc using the reductive methylation method. The GPS-MSP program ver.1.0 (http://msp.biocuckoo.org) was used to predict where the ^14^C-methyl group was labeled in the L-lysine of ^14^C-rhIL-7-hyFc. In this prediction, the entire sequence of ^14^C-rhIL-7-hyFc was examined, encompassing the Fab (IL-7 region), hyFc (IgD (hinge-CH2) and IgG4 (CH2-CH3 regions). After running the program, the organism is ‘homosapiens’ and the methylation type is ‘k.all’ (k.mono, k.di, k.tri). Afterward, the amino acid sequence information of rhIL-7-hyFc was entered in enter sequence in FASTA format, and prediction was performed at a threshold of 0.85.

## RESULTS

### PKs of serum TRA and rhIL-7-hyFc in rats

**Figure 1:**
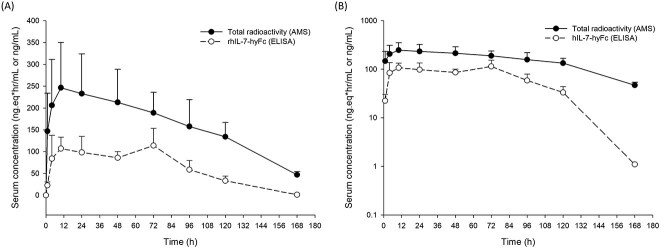
Mean serum concentration versus time of rhIL-7-hyFc and TRA in rats. TRA is expressed as the nanogram equivalents of ^14^C-rhIL-7-hyFc per gram of sample. The error bars denote the standard deviations. (A) linear scale, and (B) log scale.

After a single IM administration with rhIL-7-hyFc at 1 mg/kg, the serum TRA reached the peak around 10 hours and showed a biphasic elimination curve with a terminal half-life of 40 hours. Likewise, rhIL-7-hyFc presented the mean first peak concentration occurring ~17 hours and relatively rapid elimination with a terminal half-life of 12.3 hours (Fig. 1, Table 1). The *C*_max_ (250.8 ± 98.9 ng∙eq/ml) and AUC_last_ (27,484.1 ± 4,480.2 ng∙eq∙hour/ml) of TRA were > 2 times larger than those of rhIL-7-hyFc (*C*_max_: 123.5 ± 31.2 ng/ml, AUC_last_: 10,803.3 ± 1,484.8 ng∙hour/ml), respectively. While the terminal half-life of TRA was three times longer than that of rhIL-7-hyFc, the mean residence time was similar between TRA (67.3 hours) and rhIL-7-hyFc (59.0 hours) probably because the decrease in serum concentrations of rhIL-7-hyFc occurred toward the terminal phase, particularly at and beyond 120 hours post-dose. The apparent volume of distribution (*Vz*/*F*) of TRA and rhIL-7-hyFc were 432 and 364 ml, respectively, which were 54 times and 46 times, respectively, larger than a typical plasma volume of ~ 8 ml for a 200 g weighted-rat [9].

**Table 1:** PK parameters of TRA and rhIL-7-hyFc after a single IM administration of ^14^C-rhIL-7-hyFc (0.3285 kBq or 8.8878 nCi) at 1 mg/kg in rats (*N* = 4)

Parameter	TRA (ng.eq/ml)	rhIL-7-hyFc (ng/ml)
Analytical method	AMS	ELISA
*T*_max_ (hours)	10 (10, 24)	17 (4, 24)
*C*_max_ (ng-eq/ml or ng/ml)	250.8 ± 98.9	123.5 ± 31.2
AUC_last_ (ng-eq·hour/ml or ng·hour/ml)	27,484.1 ± 4,480.2	10,783.9 ± 1,484.3
AUC_inf_ (ng-eq·hour/ml or ng·hour/ml)	30,228.5 ± 4,687.5	10,803.3 ± 1,484.8
*t*_1/2_ (hours)	40.5 ± 8.6	12.3 ± 1.0
MRT (hours)	67.3 ± 9.3	59.0 ± 5.2
CL/F (ml/hour)	7.4 ± 0.7	20.6 ± 1.8
*Vz*/*F* (ml)	432.4 ± 105.8	364.3 ± 39.3

Mean ± standard deviation is shown except for *T*_max_, presented as the median (minimum, maximum). AUC_last_, area under the serum concentration–time curve from time point 0 hour to the last measurable concentration; AUC_inf_, area under the serum concentration-time curve from time point 0 hour to infinity; *C*_max_, initial peak serum concentration; *T*_max_, time to initial peak serum concentration after IM administration; *t*_1/2_, terminal half-life; CL/F, apparent systemic clearance from serum after IM administration; *V*/*F*, volume of distribution during the terminal phase after IM administration.

### Tissue distribution of TRA

**Figure 2:**
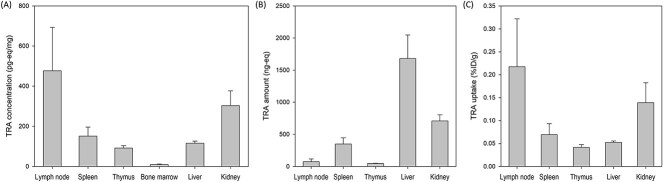
Mean tissue distribution of TRA after a single IM administration of ^14^C-rhIL-7-hyFc (0.3285 kBq or 8.8878 nCi) at 1 mg/kg in rats (*N* = 4). The error bars denote the standard deviations. (A) TRA concentration, (B) TRA amount, and (C) TRA uptake.

The mean tissue TRA concentration at 168 hours post-dose was highest in the lymph node (477.1 pg·eq/mg), followed by the kidney (303.1 pg·eq/mg) and spleen (151.1 pg·eq/mg). On the other hand, the total organ TRA amount was greatest in the liver (1,680.4 ng·eq), followed by the kidney (708.6 ng·eq). As a consequence, the mean organ TRA uptake, i.e., the ratio of the total TRA per gram of each organ to the administered radioactivity, showed the largest value in the lymph node (0.22 %ID/g), followed by that of the kidney (0.14 %ID/g), spleen (0.07 %ID/g), liver (0.05 %ID/g), and thymus (0.04 %ID/g) (Fig. 2, Table 2).

**Table 2:** Tissue distribution data after IM administration of single dose of ^14^C-rhIL-7-hyFc (0.3285 kBq) at 1 mg/kg rhIL-7-hyFc

**Tissue**	**Lymph node**	**Spleen**	**Thymus**	**Bone marrow**	**Liver**	**Kidney**
TRA concentration (pg·eq/mg)	477.1 ± 216.4	151.1 ± 45.0	91.5 ± 12.4	9.5 ± 3.1	115.8 ± 10.4	303.1 ± 73.8
TRA amount (ng)	74.3 ± 42.4	349.7 ± 95.0	46.1 ± 5.3	—	1680.4 ± 367.1	708.6 ± 95.7
TRA uptake (%ID/g)	0.22 ± 0.10	0.07 ± 0.02	0.04 ± 0.01	—	0.05 ± 0.00	0.14 ± 0.04

Data were presented as mean ± standard deviation. TRA amount and TRA uptake in bone marrow were not estimated due to the unmeasurable weight of bone marrow.

### Simulation of ^14^C labeling position

To simulate the residue where ^14^C was labeled in rhIL-7-hyFc, the number of ^14^C per one molecule of rhIL-7-hyFc and the number of L-lysine to which ^14^C-methyl-group could be labeled were investigated. The number of ^14^C per one molecule of rhIL-7-hyFc was 2–2.6 based on the SA of ^14^C-rhIL-7-hyFc. According to the sequence of rhIL-7-hyFc, it was identified that the number of L-lysine was 50 on the surface and 28 on the internal side. The GPS algorithm of GPS-MSP program calculates a score to evaluate the potential of methylation. The positions of L-lysine where ^14^C was labeled through methylation resulting in ^14^C-rhIL-7hyFc were estimated at the 13th (score 2.76), 31st (score 24.5), 71st (score 5.55), and 123rd (score 23.39) of the amino acid sequence. The higher the score, the higher the possibility of methylation of the residue. Therefore, the position of L-lysine where the ^14^C-methyl-group is more likely to be labeled in ^14^C-rhIL-7-hyFc was the 123rd and 31st residues, which were located in the IL-7 protein of rhIL-7-hyFc (Fig. S1).

## DISCUSSION

We evaluated the PK profile and disposition of ^14^C-labeled rhIL-7-hyFc or an Fc-fused rhIL-7-hyFc in rats after IM administration using two quantitative bioanalytical methods, i.e., ELISA and AMS. A ligand-binding assay, including ELISA, has been widely used to quantify a biologic product because it provides high sensitivity, specificity, and ease of use at a relatively low cost. However, its specificity and accuracy can be reduced if the analytes of interest are modified or degraded, which can be further complicated by interference by ADA. The performance of the ELISA can be also affected by matrix effects and inter-species differences [10, 11]. AMS is an emerging quantitative tool, which is useful not only for chemical compounds but also for protein pharmaceuticals in early drug development due to its high sensitivity [12–14]. Additionally, AMS is a novel method for the absolute quantification of a therapeutic protein, including mAbs and Fc-fusion proteins [11, 15]. Furthermore, AMS neither needs internal standard nor is affected by matrix effect [12, 16]. Nanotracing combined with AMS can provide correct quantification with a very small amount of sample [12], which helps alleviate many safety concerns. Additional structure analysis of ^14^C-labeled biologics help us to apprehend various analyte forms existing *in vivo* and distinguish exogenous and endogenous substances. However, manufacturing of tracer-labeled protein pharmaceuticals is challenging because small changes in the manufacturing process might affect the pharmacology and PK profiles [12]. An integrative approach using two different bioanalytical methods as adopted in this study may enabled us to improve our understanding on the PK profile and disposition of an ABT. As antibody engineering technology advances, various types of ABT products, such as mAbs, Fc-fusion proteins, and ADCs, are emerging. These products possess complex structures with distinct characteristics and functions associated with each structure, significantly influencing PK and PD. Therefore, the quantification and comprehensive interpretation of all forms that can exist in the body become crucial elements for enhancing the understanding of the PKPD properties of drugs and for successful development. For instance, in the case of ADCs, a widely developed class of ABTs, the product is a combination of an antibody and a small-molecule compound. The complimentary analytic methods applied in this study can also prove to be valuable in determining the ADME of such products.

**Figure 3:**
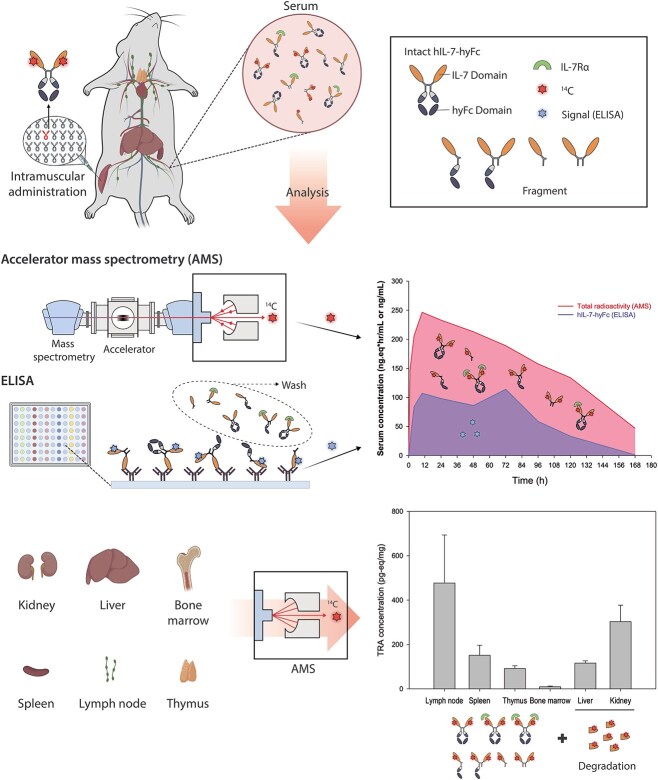
Summary of the complimentary approach using both AMS and ELISA to evaluate PK journey of rhIL-7-hyFc.

In this study, the peak concentration and AUC_last_ of TRA was 2 and 2.7 times greater than those of serum rhIL-7-hyFc, respectively (Fig. 1, Table 1). The difference in the systemic exposure to TRA and rhIL-7-hyFc is most likely to be attributed to the diversity of the analytes targeted by each bioanalytical method. For example, the target analytes for AMS are any moiety containing ^14^C. They include free ^14^C-rhIL-7-hyFc, partially free ^14^C-rhIL-7-hyFc, ^14^C-rhIL-7-hyFc with both IL-7 bound to the IL-7 receptor (IL-7R), ^14^C-IL-7 in the rhIL-7 portion, and fragments having only ^14^C-methyl groups. Because rhIL-7-hyFc is stable in various stress conditions, a significant portion of TRA is likely to contain free or partially free, bound forms of intact rhIL-7-hyFc (Fig. 3). On the other hand, serum rhIL-7-hyFc concentration determined by ELISA is more likely to represent the level of free or partially free forms of rhIL-7-hyFc. Even if one molecule of rhIL-7-hyFc has two unbound rhIL-7 portions, it is assumed that only one rhIL-7 was measured by binding to ligand due to structural interferences. Moreover, TRA showed significant differences in half-life (31.9–59.1 hours) and CL/F (7.4 ml/hour) from those of rhIL-7-hyFc (12.3–25.6 hours and 20.6 ml/hours, respectively) in this study (Table 1) and the previous nonclinical study (data not shown). These findings can be understood by linking them to the clearance difference between tissues observed in the PBPK model of antibody [17]. The PBPK model provided a systematic quantification of clearances specific to different tissues and highlighted the significance of FcRn protection in each individual tissue [17]. The extended elimination and low clearance observed in TRA are speculated to be a result of the measured fragments degraded by tissues with high intrinsic clearance, such as the liver, muscles, spleen, and other tissues. This is an observable result because AMS can measure analytes that exist in various forms at all stages of ADME with high sensitivity.

After the IM administration of high molecular weight biotherapeutics, it is primarily distributed by the diffusion or convection from the injection site to the systemic circulation via mainly lymphatics and from the blood compartment to peripheral tissues [18–21]. The large size and hydrophilic nature of ABTs generally lead to a low volume of distribution, approximately similar to the plasma volume [18]. However, in this study, the apparent volume (*Vz*/*F*) of TRA and rhIL-7-hyFc were 432 and 364 ml, respectively, which are much larger than the total plasma volume of a rat weighing 200 g, i.e.,~8 ml [9]. This discrepancy was likely because rhIL-7-hyFc and its drug-related material, i.e., those detected as TRA, can move to their target organs and tissues, mostly via lymphatics, because they have a high affinity for the target ligand. To support this notion, the mean TRA concentration and TRA uptake at various organs were relatively high, particularly in the lymphoid organs, including the lymph node and spleen (Fig. 2, Table 2). The important role of lymphatic system in the absorption of biotherapeutics accounts for these findings [20]. Additionally, based on the previous reports showing common responses in mice, non-human primates, and humans after the administration of the recombinant human IL-7 [22–24], those findings suggest rhIL-7-hyFc is relatively well distributed to various target tissues after IM administration in humans. IL-7R is composed of two chains IL-7Rα, which is exclusively expressed on lymphoid cells [22]. The lymphoid tissue made up by these lymphoid cells is found in the lymph node, spleen, and thymus. These tissues expressing IL-7Rα might contribute to the large apparent volume of distribution of rhIL-7-hyFc. A similar finding was also noted in the FIH study with rhIL-7-hyFc [7], where the apparent volume of distribution of rhIL-7-hyFc in humans was 4578 l after a single IM injection at 60 μg/kg, which is way greater than the typical human blood volume of ~ 5 l. Likewise, the RNA expression of IL-7R was remarkably high in the bone marrow and lymphoid tissues according to the Human Protein Atlas Project [25].

In this study, the other organs showing high TRA uptake and TRA concentrations were the spleen, kidney, and liver, which are highly vascularized organs. Shah *et al*. estimated a similar value of the antibody distribution coefficient (ABC) for the kidney (13.7%), spleen (12.8%), and liver (12.1%) based on a physiologically based PK model of mAb [26]. An organ ABC is the ratio of the concentration in the organ to plasma assuming a linear relationship between the plasma and tissue concentration of non-binding mAb [26]. Likewise, the mean tissue TRA concentrations in the lymph node, kidney, spleen, and liver were high at 115.8–477.1 pg eq/mg in this study (Table 2). Taken together, our findings support the mechanism of action rhIL-7-hyFc. Specifically, the TRA concentration in the lymph nodes was observed to be exceptionally higher compared to other tissues, more drastically than the previous research (ABC: 8.46) [26]. This is determined to be associated with rhIL-7-hyFc’s mechanism of action along with not only the process of filtering immune cells in the lymphatic vessels but also the distribution process after the IM administration. Furthermore, the spleen allows high molecular proteins to enter its parenchyma due to its high blood flow and loose capillaries and to bind the target expressed in cell population of the spleen. Thus, rhIL-7-hyFc not only may bind to IL-7R expressed in the spleen but also can enter the parenchyma of the spleen, resulting in various biological effects. Furthermore, the liver and kidney are also play a role in the metabolism and elimination of mAbs [18]. The liver contributes to the elimination of IgG and proteolysis of mAbs [18]. The catabolized mAb, i.e., fragments consisting of peptides and amino acids can be excreted via the kidney if they are not greater than a molecular weight of 50 kDa. The relatively higher TRA concentration and TRA uptake in the kidney noted in this study also suggest the drug-related materials in the kidney were most likely degraded or catabolized fragments of rhIL-7-hyFc rather than its intact forms.

This study had a few limitations. Most notably, we studied the PK profile and disposition of rhIL-7-hyFc only in rats. However, human IL-7 has been confirmed to be effective in mouse, rat, monkey, and human. Therefore, although further studies are warranted to evaluate if the findings in this study are extrapolated to other species, the insights gained from the present study are still useful to better understand the role of rhIL-7-hyFc in humans. Second, we did not assess the distribution of TRA for rhIL-7-hyFc to other tissues than the target organs such as lymphoid tissues and elimination organs. However, given the high specific nature of target-binding for ABTs, such as rhIL-7-hyFc, the disposition of drug-related material to other unstudied organs and tissues could have been minimal, if not none. Lastly, given the limitations of the organ-specific TRA values observed in this study, which do not directly provide information about the form of the drug in each organ, the interpretation of the results was based on the considerations of the simulated ^14^C residue positions, the PK properties of rhIL-7-hyFc, and the physiological characteristics of each organ. This interpretation allows for an inference regarding the significance of the results. If further basic research on the PK properties of ABTs is conducted, this explanation can be reexamined and replenished.

In conclusion, using AMS and ELISA in a complementary way, we were able to better understand the PK profile and disposition of rhIL-7-hyFc in rats. Furthermore, we showed that ^14^C-rhIL-7-hyFc is relatively highly distributed to the lymphoid and eliminating organs, the latter of which could probably account for the degraded or catabolized drug-related materials of rhIL-7-hyFc. Our results can shed light on the PK fate of ABTs, mostly unknown or less studied so far.

## Supplementary Material

Mab_Supplementary_Data_20231020_tbae004(1)

## Data Availability

The data that support the findings of this study are available within the article and supplementary information. Additional data will be shared on reasonable request to the corresponding authors.

## ANIMAL RESEARCH

The study was performed according to the guidelines and with the approval of the IACUC of the KIST (IACUC number: KIST-2019-022).

## CREDIT TAXONOMY

Anhye Kim (Conceptualization [lead], Data curation [equal], Formal analysis [equal], Funding acquisition [lead], Investigation [lead], Methodology [lead], Project administration [equal], Resources [supporting], Supervision [lead], Validation [supporting], Visualization [equal], Writing—original draft [lead], Writing—review & editing [lead]), Min-Seok Oh (Conceptualization [equal], Data curation [equal], Formal analysis [equal], Investigation [equal], Methodology [equal], Project administration [supporting], Resources [equal], Validation [equal], Visualization [equal], Writing—original draft [equal], Writing—review & editing [equal]), Gwan-Ho Lee (Conceptualization [equal], Data curation [equal], Formal analysis [equal], Investigation [equal], Methodology [equal], Resources [equal], Software [equal], Validation [equal], Visualization [equal], Writing—original draft [supporting], Writing—review & editing [supporting]), Seongeun Song (Data curation [supporting], Formal analysis [supporting], Investigation [supporting], Resources [supporting], Validation [supporting], Visualization [supporting], Writing—original draft [supporting], Writing—review & editing [supporting]), Mi-sun Byun (Resources [supporting], Validation [supporting], Writing—review & editing [supporting]), Donghoon Choi (Investigation [supporting], Resources [supporting], Writing—review & editing [supporting]), Byung-Yong Yu (Conceptualization [lead], Data curation [lead], Formal analysis [lead], Investigation [lead], Methodology [lead], Project administration [equal], Supervision [lead], Validation [lead], Visualization [equal], Writing—original draft [supporting], Writing—review & editing [equal]), and Howard Lee (Conceptualization [equal], Funding acquisition [lead], Investigation [equal], Methodology [equal], Project administration [lead], Resources [lead], Supervision [lead], Writing—original draft [supporting], Writing—review & editing [equal]).
