# Supplementary material for: Understanding the pharmacokinetic journey of Fc-fusion protein, rhIL-7-hyFc using complementary approach of two analytical methods, accelerator mass spectrometry and ELISA
Source: Antib Ther. 2024 Feb 6;7(2):105–13. doi: 10.1093/abt/tbae004 (PMC10983079; doi:10.1093/abt/tbae004)
Supplement: Mab_Supplementary_Data_20231020_tbae004(1) [file mab_supplementary_data_20231020_tbae004(1).docx]

Figure S1. Model structures of rhIL-7-hyFc use the IL-7 (PDB 3DI3) and Phyre v2.0 [1] obtained hyFc model using the IgG4 Fc as template (PDB 4C54). Model building using COOT [2] and figures were prepared using Pymol [3]. Left is surface model; Green: IL-7, Orange/Yellow orange: hyFc dimer. Right is electrostatic potential surfaces model; Red: negative charge, Blue: positive charge, white: nonpolar charge.


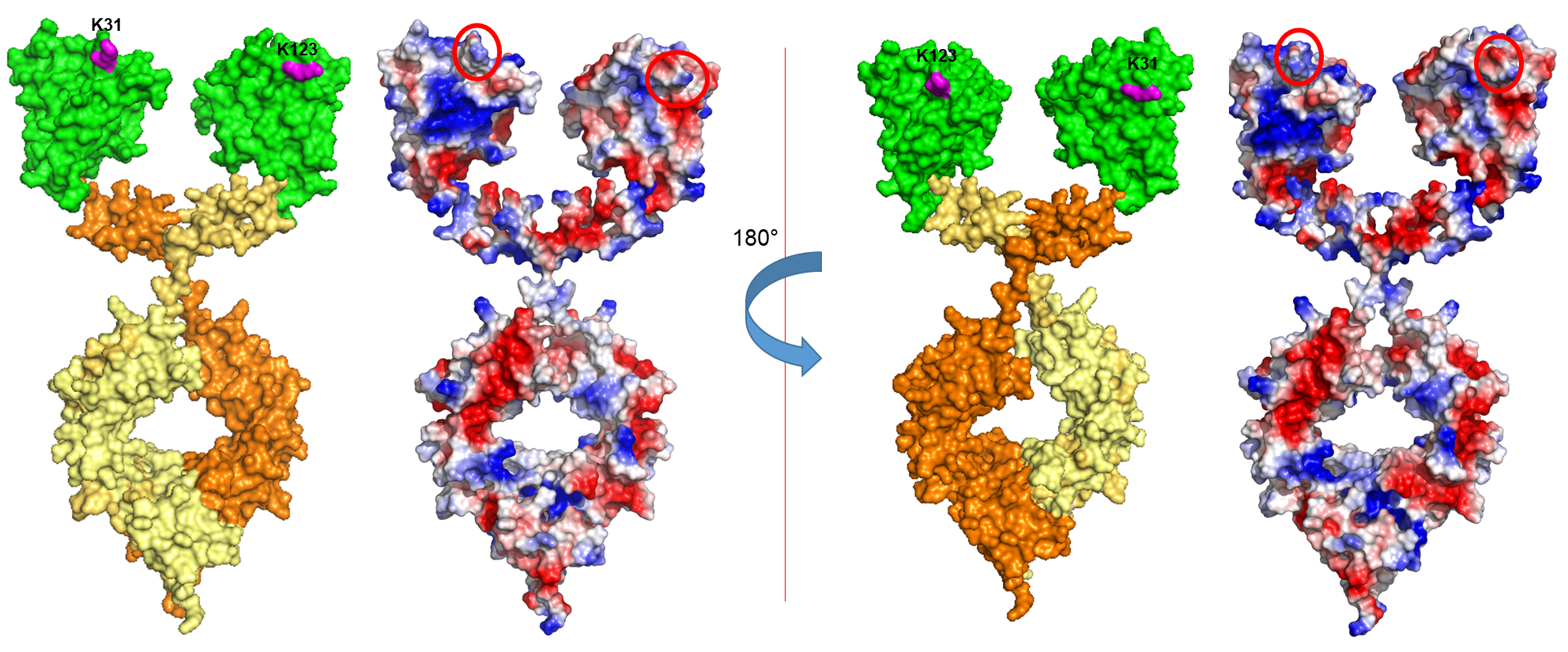


**References**

1. Kelley, LA, Sternberg, MJ. Protein structure prediction on the web: a case study using the Phyre server. *Nat Protoc* 2009; **4**: 363–71.

2. Emsley, P, Cowtan, K. Coot: model-building tools for molecular graphics. Acta Crystallogr D Biol Crystallogr 2004; 60: 2126–32.

3. Schrodinger, LLC. The PyMOL Molecular Graphics System, Version 2.0. 2015. https://pymol.org/ (accessed 2023 Jan 3).
